# The Basal or Sphenopetrosal Superficial Middle Cerebral Vein Type

**DOI:** 10.3390/medicina60020209

**Published:** 2024-01-25

**Authors:** Adelina Maria Jianu, Monica Adriana Vaida, Mugurel Constantin Rusu, Alexandra Diana Vrapciu

**Affiliations:** 1 Department of Anatomy and Embryology, Faculty of Medicine, ”Victor Babes” University of Medicine and Pharmacy, 300041 Timisoara, Romania; adelina.jianu@umft.ro (A.M.J.); vaida.monica@umft.ro (M.A.V.); 2 Division of Anatomy, Faculty of Dentistry, “Carol Davila” University of Medicine and Pharmacy, 050474 Bucharest, Romania; alexandra.vrapciu@umfcd.ro; 3 University Emergency Hospital Bucharest, 050098 Bucharest, Romania

**Keywords:** skull base, Sylvian vein, Sylvian fissure, temporal bone, TMJ, tegmen tympani, middle cranial fossa

## Abstract

*Background and Objectives*: The adult superficial middle cerebral vein (SMCV) commonly drains into the middle cranial fossa. However, different embryonic types persist, in which the SMCV drains into the lateral sinus. The basal type of SMCV coursing on the middle fossa floor is a scarce variant. *Materials and Methods*: During a retrospective study of archived computed tomography angiography (CTA) and magnetic resonance angiography (MRA) files, three rare adult cases of the basal or sphenopetrosal type of SMCV were found and further documented. *Results*: In the first case, which was evaluated via CTA, the basal type of SMCV formed a sagittal loop. It continued on the middle fossa floor, over a dehiscent tegmen tympani, to drain into the lateral sinus. In the second case, documented via MRA, the basal type of SMCV’s anterior loop was in the coronal plane and closely related to the internal carotid artery and the cavernous sinus. It continued with the basal segment over a dehiscent glenoid fossa of the temporomandibular joint (TMJ). In the third case, documented via CTA, the initial cerebral part of the SMCV had a large fenestration. The middle fossa floor coursed within a well-configured sulcus of the SMCV and received a tributary through the tympanic roof. Its terminal had a tentorial course. *Conclusions*: Beyond the fact that such rare variants of the SMCV can unexpectedly interfere with specific approaches via the middle fossa, dehiscences of the middle fossa floor beneath such variants can determine otic or TMJ symptoms. Possible loops and fenestrations of the SMCV should be considered and documented preoperatively.

## 1. Introduction

Cerebral veins are classified into deep and superficial interconnected groups [1]. Superficial cerebral veins drain cortical surfaces and subcortical white matter [1,2]. Countless morphologic variations in surface venous anatomy can be conceptualized as a balance between the four main collectors: superior and inferior (Labbé’s) anastomotic veins, the superficial middle cerebral vein (SMCV) or superficial Sylvian vein, and the basal vein [3]. The progressive dominance of one vein or co-dominance is usually associated with corresponding hypoplasia of the other veins [3,4].

The superficial middle cerebral vein (SMCV) usually runs anteriorly and inferiorly to the pterional region along the Sylvian fissure to drain into either the sphenoparietal sinus or the cavernous sinus [5,6,7]. During development, the SMCV does not drain into the cavernous sinus, but into an embryonic dural channel (the tentorial sinus of Padget), which further joins the lateral sinuses (transverse and sigmoid sinuses) [8]. The SMCV can also drain, at least partly, into the middle meningeal vein, the vein in the foramen lacerum, the superior sagittal sinus, or the superior petrosal sinus [9,10]. Commonly, the SMCV is connected to the transverse sinus (TS) by the vein of Labbé [6]. Hacker (1974), quoted by Shibao et al. (2016), classified the SMCV into four types: cavernous, emptying into the cavernous sinus; sphenobasal, coursing through the foramen ovale; sphenopetrosal, draining into the lateral sinus; and absent [7,11].

The endovascular approach has evolved as the mainstay therapy for definitively treating carotid–cavernous sinus fistulas [12]. Carotid–cavernous sinus fistulas are abnormal communications between the internal carotid artery system and the cavernous sinus [12]. The anatomic complexity of the cavernous sinus makes the endovascular treatment of indirect carotid cavernous fistulas the preferred method of treatment [13]. Some of such fistulas may benefit from transvenous embolizations via multiple routes, the inferior petrosal sinus being preferred [12]. The cavernous sinus may also be accessed via the Labbé vein or the SMCV [13]. Therefore, anatomic variations of the SMCV should be assessed, especially in dural arteriovenous fistulas with cortical venous congestions [14].

A case series with a rare anatomic variant, the basal type of SMCV, is reported here.

## 2. Materials and Methods

In three archived cases retrospectively anatomically evaluated, basal types of SMCV were found. The first and third cases were scanned via computed tomography angiography (CTA). The second case was examined via magnetic resonance angiography (MRA).

The anatomic variants in cases 1 and 3 were found during a retrospective computer tomography study in a 58 y.o. male’s case and a 50 y.o. male’s case, respectively. Examinations were performed using a 64-slice CT Somatom Definition As (Siemens, Forcheim, Germany), with a rotation time of 0.5 s, using a pitch of 1.2 and a collimation of 1.2 mm. CareDose4D and CareKV were used to reduce the radiation dose. Examinations were performed with patients in the head-first prone position, with an inspiratory breath hold. We injected a volume of 90 mL Omnipaque (300 mg I/mL) followed by a 40 mL saline bolus with a 3.5 mL/s flow. The scan start for the arterial phase was auto-triggered when the contrast in the pulmonary artery increased by 100 HU compared to the non-enhanced scan. The venous phase was performed with a delay of 30 s after the arterial phase. For primary diagnosis, 5 mm and 3 mm reconstructions were used with no overlap, and 1.5 mm reconstructions were used with a 0.5 mm overlap and a B31f image filter for multiplanar reconstructions, maximum intensity projection, and volume rendering images.

The anatomic variant in case 2 was found when documenting the archived MRA files of a 66 y.o. female’s case. The MR examination was performed using a 3T Magnetom Vida system (Siemens) using a Head Neck 20TCS coil. The scan protocol included the following sequences: t1_fl2d_sag_4mm, t2_tse_tra_512_4mm, t2_tse_dark-fluid_tra_3mm, resolve_4scan_trace_tra_p2_192_4mm, t2_swi_tra_p2_2mm, t1_fl2d_tra_4mm, t2_tse_dark-fluid-cor_3mm, and t1_mprage_tra_p2_iso_0.9mm. We injected a volume of 20 mL Clariscan followed by a 20 mL saline bolus with a flow of 3 mL/s and repeated t1_mprage_tra_p2_iso_0.9mm (with automatic subtraction of the non-enhanced sequence), followed by t1_se_tra_blood-suppr._MTC_4mm. MPR and volumetric reconstruction were performed using the t1_mprage sequence.

Anatomic variants were documented using the Horos 4.0.1 for Apple Silicon software (Horos Project). Research was ethically conducted following The Code of Ethics of the World Medical Association (Declaration of Helsinki). Subjects gave their informed consent for inclusion before participating in this study, and the responsible authorities (affiliation 3) approved this study (approval no. 10540/16.02.2022).

## 3. Results

**Figure 1:**
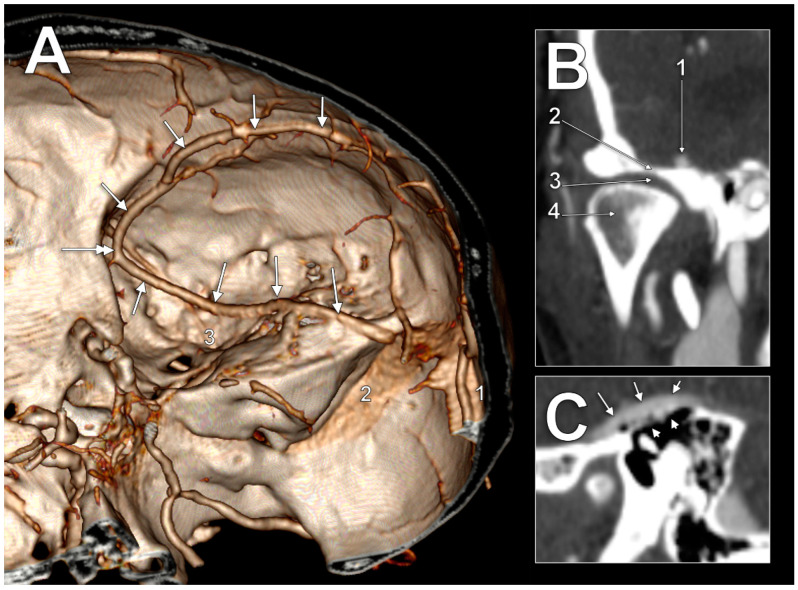
(**A**) CT angiography, three-dimensional volume rendering of the right superficial middle cerebral vein (SMCV) of the basal type (arrows). Superomedial view. 1. transverse sinus; 2. sigmoid sinus; 3. foramen spinosum. The sagittal loop of the SMCV is indicated (double-headed arrow). (**B**) Coronal CT slice through the right temporomandibular joint. 1. superficial middle cerebral vein; 2. mandibular fossa; 3. articular disc; 4. mandibular condyle. (**C**) Sagittal CT slice through the right tympanic cavity. The superficial middle cerebral vein (arrows) courses on a dehiscent tympanic roof (arrowheads).

In the first case (Figure 1A), the SMCV had an initial postero–anterior course, superficially, in the Sylvian fissure. At the pterion level, it continued descending on the temporal lobe to until it was posterior to the lateral part of the superior orbital fissure, where it formed a sagittal loop on the medial side of the anterior branch of the middle meningeal artery to further continue anteroposteriorly on the middle cranial fossa floor. In the middle fossa, it had four segments: (1) an alar segment on the greater sphenoidal wing; (2) a squamosal segment on the horizontal part of the temporal squama; (3) a petrosal segment on the petrous part of the temporal bone; and (4) a mastoid segment, on the mastoid part of the temporal bone. The alar segment coursed at 1.1 cm lateral to the foramen ovale and at 5.1 mm lateral to the foramen spinosum and the entry of the middle meningeal artery. Then, it continued as the squamosal segment. The squamosal segment was first located above the articular eminence, then above the inner part of the mandibular fossa (Figure 1B) above the temporomandibular joint. The following petrosal segment coursed above the tympanic cavity at 3.4 mm from the head of the malleus. The tympanic roof (tegmen tympani) was thinned/dehiscent (Figure 1C). The last mastoid segment coursed obliquely above the mastoid air cells to posteromedially drain into the junction of the right transverse and sigmoid sinuses.

**Figure 2:**
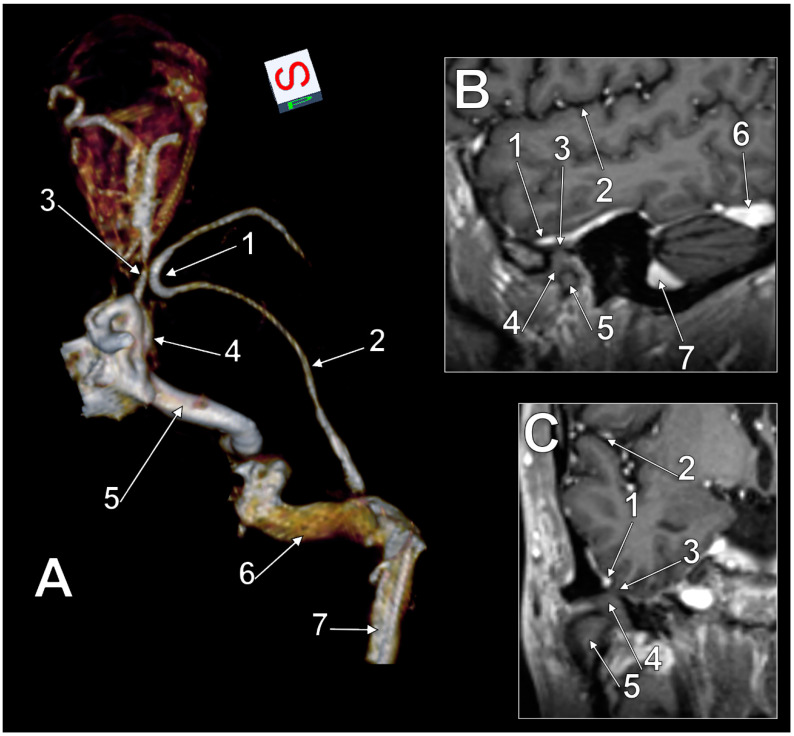
Three-dimensional volume rendering of (**A**) the right superficial middle cerebral vein (SMCV). Postero-supero-medial view. 1. coronal loop of the SMCV; 2. basal course of the SMCV; 3. superior ophthalmic vein; 4. cavernous sinus; 5. internal carotid artery; 6. sigmoid sinus; 7. transverse sinus. (**B**) Sagittal and (**C**) coronal MRI slices through the right temporomandibular joint. 1. superficial middle cerebral vein of the basal type; 2. Sylvian fissure; 3. dehiscent mandibular fossa of the temporal squama; 4. articular disc; 5. mandibular condyle; 6. transverse sinus; 7. sigmoid sinus.

In the second case (Figure 2A), the right SMCV began in the Sylvian fissure and then coursed anteriorly on the lateral side of the temporal lobe until it was posterior to the superior orbital fissure. It then formed a medially convex large coronal loop around the temporal pole. The convexity of the SMCV coronal loop contacted the superior ophthalmic vein; it was at a 4.8 mm distance from the internal carotid artery and 1.9 mm from the cavernous sinus. Then, this SMCV continued inferolaterally, crossed the maxillary nerve superiorly at 2.5 mm, and reached the middle cranial fossa floor. The basal course of this SMCV had, as in the previous case, alar, squamous, petrous, and mastoid segments. This SMCV ended in the junction of the right transverse and sigmoid sinuses. As the right mandibular fossa was dehiscent, the squamous basal segment of this SMCV was closely related to the temporomandibular joint disc (Figure 2B,C). The vein was just 1.05 mm above that disc, these two being separated by only the dura mater.

**Figure 3:**
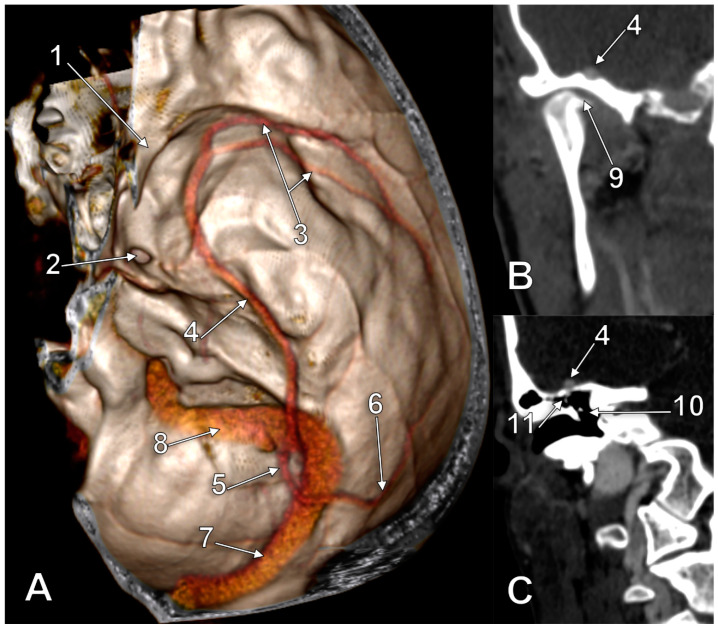
Three-dimensional volume rendering of (**A**) a right-fenestrated superficial middle cerebral vein (SMCV) of the basal type, with a tentorial terminal end. (**B**) Coronal slices through the temporomandibular joint (TMJ) and (**C**) tympanic cavum. 1. lesser sphenoidal wing; 2. foramen ovale; 3. large fenestration of the SMCV; 4. SMCV on the middle fossa floor; 5. tentorial terminal segment of the SMCV; 6. vein of Labbé; 7. transverse sinus; 8. sigmoid sinus; 9. TMJ; 10. tympanic cavum; 11. tympanic tributary of the SMCV.

In the third case (Figure 3), the SMCV formed in the Sylvian fissure. Its first segment had a large fenestration, 4.21 cm in length and 0.87 cm in width. The pterion centred the fenestration. The basal course of the unique trunk of this SMCV had alar, squamous, petrous, and mastoid segments. The alar segment was at 1.85 cm lateral to the foramen rotundum and at 1.36 cm lateral to the foramen ovale. Then, it continued over the horizontal part of the temporal squama in a groove extending posteriorly over the tympanic cavum and mastoid antrum. The anterior end of the sulcus of the SMCV was at 1.35 cm lateral to the foramen of Arnold, which, in turn, was at 0.66 cm posterior to the foramen spinosum. On the tympanic roof the SMCV received a tributary from the tympanic cavum. The SMCV finally crossed the petrous ridge and continued with a terminal tentorial segment that joined the lateral end of the transverse sinus at the same point as the Labbé’s vein did on that side.

## 4. Discussion

Although the SMCV, also called the superficial Sylvian vein or Sylvian vein, is typically described as terminating into the cavernous sinus, different anatomical possibilities for SMCV drainage were previously found (see the Section 1). They were consistently documented by Bisaria in 1985 [9]. Different variations can be assigned to SMCVs [15]; thus, a SMCV variant should not be unexpected. A SMCV is present in 90% of cases [15]. A SMCV can also be hypoplastic, consisting of a single main stem draining into the sphenoparietal sinus, or two main stems draining into the sphenoparietal sinus [15]. Here, we contribute overlooked details of the basal course of the SMCV. To our knowledge, neither fenestrations nor loops of a basal SMCV have been previously reported.

During embryonic development, intracranial venous channels undergo significant anatomical changes [16]. The SMCV results from the primitive tentorial sinus that superiorly and posteriorly drains into the primitive marginal sinus [16]. During development, the drainage pattern shifts from the primitive tentorial sinus to the cavernous sinus [16]. Thus, the drainage route of the SMCV shifts from a posterior one to a medial one. Primitive phenotypes of drainage can persist in adults as variants of the SMCV (sphenoparietal, cavernous, emissary, superior petrosal, basal, or squamosal) [5,6,14]. The basal type of SMCV, when the vein turns downward along the floor of the middle cranial fossa and runs over the petrous bone to enter the transverse sinus, was found in just 2% of 500 SMCVs [5]. The downward turn of the SMCV is the sagittal loop of the SMCV we saw in the first case. However, a different coronal loop of the SMCV was found in the second case. The third case was quite similar to the first one, with several differences: the large fenestration of the SMCV, the basal sulcus of the SMCV, and the tentorial type of termination.

To the authors’ knowledge, fenestrations of the SMCV have not been previously reported. Neither has the basal sulcus of the SMCV crossing over the tegmen tympani, which is anatomically different than a possible petrosquamous sulcus located on the petrosquamous suture and, therefore, located more internally.

Matsushima et al. (1989) classified tentorial sinuses into four groups: group I, in which the sinus receives blood from the cerebral hemisphere; group II, in which it drains the cerebellum; group III, in which the sinus originates in the tentorium, and group IV, in which the sinus arises from a vein bridging to the tentorial free edge [17]. The tentorial termination of the SMCV thus corresponds to the first type of tentorial sinus. Neurosurgeons are particularly interested in tentorial sinuses because they represent obstacles during surgical procedures, such as transoccipital transtentorial, infratentorial supracerebellar, and subtemporal transtentorial approaches [17].

Hacker (1974), quoted by Shibao et al. (2016), classified the SMCV into four types: sphenoparietal sinus, sphenobasal vein, sphenopetrosal vein, and absent SMCV [7,11]. The sphenopetrosal type of SMCV corresponds to the basal type indicated by other authors and us [5,14,16]. Shibao et al. (2016) described three subtypes of the sphenopetrosal SMCV: vein, vein–sinus, and sinus. For each of these subtypes, specific modifications of the anterior petrosectomy are recommended [7]. The first case we report here corresponds to the 3a (vein) subtype of Shibao, which occurs in 14.6% of cases [7]. The third case we report corresponds to the 3b (vein–sinus) subtype of the sphenopetrosal SMCV in Shibao’s classification, which occurs in 2.1% of cases [7].

The second case we report here presents a peculiar morphology—looped SMCV—that, to our knowledge, has not been previously associated with the sphenopetrosal type. Wolf et al. (1963), quoted by Bisaria (1985), objectivated the course of a SMCV along the lesser sphenoidal wing towards the cavernous sinus [9,18]. This corresponds to the location of the sinus of the lesser sphenoidal wing detailed by San Milan Ruiz et al. (2004). These authors regarded the sphenoparietal sinus as the artificial combination of two venous structures: the parietal segment of the anterior branch of the middle meningeal vein and the sinus of the lesser sphenoidal wing [19]. Notably, the sphenoparietal sinus was equally regarded as the superior sphenoidal sinus, sinus alae parvae, or the sinus of Breschet [20]. In a dissection study quoted by Bisaria (1985), Parkinson (1965) presented evidence for the “dural opening for the Sylvian vein group into the superior sphenoidal sinus” [21]. However, that evidence may be regarded as just a cavernous sinus drainage pattern, on the one hand, and on the other hand, does not convincingly demonstrate that those veins were indeed SMCVs, as the brain was removed entirely from the skull base [21]. In the second case we report here, the upper arm of the coronal loop of the SMCV corresponds to a lesser wing level of that vein’s course.

Cerebral veins may be damaged during operations by three mechanisms: intentional coagulation and division to prevent their rupture, traction, during an operation, from their fixed drainage sites (into a dural sinus) causing their rupture, and damage during their dissection in the brain [22]. Neurosurgical approaches most commonly used to access supratentorial structures are the pterional and orbitozygomatic approaches. Using such approaches, the middle cerebral artery can be approached. Despite careful surgical dissection, placement of retractors, and separation of brain structures, the SMCV may be compromised [23]. Basal operations also stretch veins and put them at risk for rupture [22]. A basal SMCV interferes with an anterior transpetrosal neurosurgical route. Extradural procedures and dural incisions during an anterior transpetrosal approach may interrupt the drainage route from the SMCV [7]. A SMCV not draining into the cavernous sinus cannot be used as an endovascular route to approach this sinus, as previously undertaken [13].

The aetiology of temporomandibular joint (TMJ) pain is not fully understood [24]. Temporal articular surfaces can be pneumatised, and these air spaces can be dehiscent upwards beneath the dura mater. The roof of the mandibular (glenoid) fossa is commonly thin. To our knowledge, perforated glenoid fossa is extremely rare and rarely reported. In such cases, the TMJ disc is directly superiorly related to the dura mater and, as we demonstrated, to a basal type of SMCV. In such cases, an increase in blood pressure or volume within the supraarticular dura mater can cause a TMJ migraine.

Before using lateral approaches to the middle cranial fossa, the anatomic possibility of a basal type of SMCV should be documented to avoid further unwanted haemorrhagic incidents.

The SMCV runs close to the internal carotid artery and cavernous sinus in the basal SMCV type with a coronal loop (second case). Therefore, specific neurosurgical approaches to the internal carotid artery and/or cavernous sinus should avoid harming such a SMCV.

Tegmen defects were previously found, unilaterally or bilaterally [25,26]. Multiple tegmen defects form a characteristic ‘honeycomb’ pattern [27], such as in the first case reported here. The incidence of tegmental defects varies from below 1% up to 34% [27]. Such tegmental defects may permit infection to ascend from the tympanic cavity to the meninges [26]. A basal course of the SMCV over such defects may situate the cerebral venous system in the direct dissemination pathways of such an infection. Rhythmic movements of the tympanic membrane without retrotympanic lesions represent an unusual otoscopic finding [25]. Such movements were recorded in cases with tegmen defects, and resulted by either transmitting dural pressure waves or by a direct connection between the cerebral mass and the ossicular chain [25]. An unusual course of a SMCV over a dehiscent tegmen is an additional pressure element. This is supported by a previous report of a patient with venous pulsatile tinnitus due to a SMCV that coursed inferiorly to the temporal lobe and protruded into the tympanum through a dehiscent anterior cortical plate of the tympanum [28].

## 5. Conclusions

Anatomical details of rare variants of the SMCV are clinically and surgically important, and modern imaging techniques can be helpful for the purpose of identifying them. Possible loops and fenestrations of the SMCV should be preoperatively considered and documented.

## Data Availability

No new data were created or analysed in this study. Data sharing does not apply to this article.
